# Fertility‐Sparing Management of HPV‐Associated FIGO IB1 Mucinous Cervical Adenocarcinoma With Signet‐Ring Cell Features: A Case Report

**DOI:** 10.1155/crog/8025579

**Published:** 2026-04-08

**Authors:** Luiz Felipe Lessa Ortiz, Amanda Lino de Faria Lessa, Rafael Dyer Rodrigues de Moraes, Renan Ribeiro e Ribeiro, Henrique Cunha Vieira, Bárbara Bomfim Muniz Moraes, Roney César Signorini Filho, Giorgio Bogani

**Affiliations:** ^1^ GAVVA Clínica-Medicina Reprodutiva e Oncofertilidade, São Paulo, Brazil; ^2^ CICAP-Hospital Alemão Oswaldo Cruz, São Paulo, Brazil; ^3^ Hospital da Mulher-Pérola Byington, São Paulo, Brazil; ^4^ IRCCS Istituto Nazionale dei Tumori di Milano, Milan, Italy

**Keywords:** cervical cancer, fertility preservation, mucinous adenocarcinoma, radical trachelectomy, sentinel lymph node, signet-ring cells

## Abstract

**Background:**

Fertility‐sparing surgery is an increasingly accepted option in carefully selected patients with early‐stage cervical cancer. Radical vaginal trachelectomy combined with sentinel lymph node (SLN) mapping offers oncologic safety while preserving reproductive potential.

**Case:**

A 29‐year‐old nulliparous woman with FIGO stage IB1 poorly differentiated HPV‐associated mucinous cervical adenocarcinoma with signet‐ring cell features underwent radical vaginal trachelectomy and bilateral laparoscopic SLN mapping using indocyanine green (ICG). No nodal metastases were identified. Surgical margins were negative, and an abdominal cervical cerclage was placed. At 24 months of follow‐up, the patient remains disease‐free and is receiving counseling regarding assisted reproductive technologies.

**Conclusion:**

Fertility‐preserving surgery may be safely performed in selected early‐stage cervical cancer patients when combined with appropriate surgical staging and close follow‐up. This case adds to the limited literature regarding the conservative management of high‐grade mucinous adenocarcinomas with signet‐ring cell morphology.

## 1. Introduction

Cervical cancer remains a major global health concern, with an estimated 662,301 new cases diagnosed worldwide in 2022, representing the fourth most common cancer among women. Notably, approximately 38.5% of cases occur in women of reproductive age, highlighting the importance of fertility‐preserving strategies in appropriately selected patients [1, 2].

For early‐stage disease, particularly FIGO stage IB1, the historical standard treatment has been type C radical hysterectomy with systematic pelvic lymphadenectomy. While this approach provides excellent oncologic control, it inevitably results in permanent loss of fertility and is associated with significant surgical morbidity related to radical parametrectomy and lymphadenectomy [1, 3–5].

In recent years, growing evidence has supported the treatment de‐escalation in carefully selected patients with low‐risk disease. The SHAPE trial demonstrated that simple hysterectomy provides oncologic outcomes comparable to radical hysterectomy in patients with low‐risk tumors ≤2 cm, negative lymph nodes, absence of lymphovascular space invasion (LVSI), and no parametrial involvement, challenging the historical paradigm of mandatory radical surgery for early‐stage cervical cancer [6].

For women who wish to preserve fertility, radical trachelectomy combined with pelvic lymph node assessment has historically been the preferred option for tumors ≤2 cm. However, the role of radical procedures is evolving, and less radical fertility‐sparing approaches, including conization with lymph node staging, are increasingly being explored in highly selected cases [3, 4, 7–9].

Accurate lymph node assessment remains a critical component of surgical staging. Over the past decade, sentinel lymph node (SLN) mapping has emerged as a reliable strategy to reduce the morbidity associated with systematic lymphadenectomy while maintaining staging accuracy. Prospective trials have played a pivotal role in validating this approach. The SENTICOL‐1 study demonstrated a high detection rate and excellent sensitivity of SLN mapping in early‐stage cervical cancer [10], while the SENTICOL‐2 randomized trial confirmed that SLN biopsy significantly reduces surgical morbidity without compromising short‐term oncologic outcomes when compared with complete pelvic lymphadenectomy [11]. More recently, the SENTIX prospective multicenter study further supported the safety of SLN mapping alone in patients with negative sentinel nodes, reporting favorable oncologic outcomes and reinforcing its role as a standard staging technique in early cervical cancer [12].

Among the available tracers, indocyanine green (ICG) fluorescence imaging has shown superior bilateral detection rates compared with traditional techniques using blue dye or radiocolloid and is now widely adopted in minimally invasive gynecologic oncology surgery [5].

Despite the increasing acceptance of fertility‐sparing approaches, careful patient selection remains essential. A tumor size greater than 2 cm, the presence of LVSI, deep stromal invasion, or lymph node metastasis are generally considered contraindications to conservative management [1, 4, 7]. Therefore, multidisciplinary evaluation is crucial to balance oncologic safety with reproductive goals [3, 9, 13, 14].

In this context, we report a rare case of HPV‐associated mucinous cervical adenocarcinoma with signet‐ring cell features (FIGO IB1) managed with fertility‐sparing surgery and SLN mapping, highlighting diagnostic challenges and considerations for conservative management in this unusual histologic subtype [2, 5, 8, 9, 14–16].

## 2. Case Report

**Figure 1:**
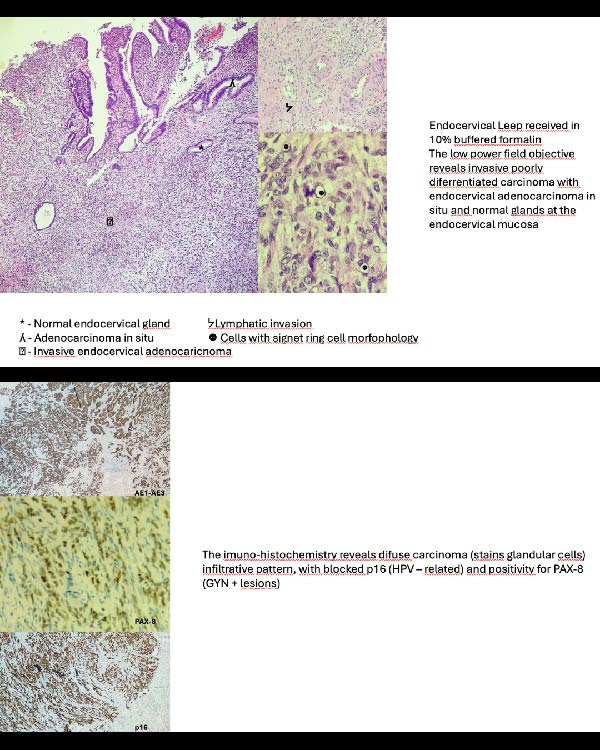
The image presents a histological evaluation of the cervical cone specimen, which revealed an invasive mucinous adenocarcinoma, and immunohistochemical staining performed at the referring institution demonstrated block‐type diffuse positivity for p16, along with strong and diffuse expression of AE1/AE3 pancytokeratins and PAX8, supporting the diagnosis.

We report the case of a 29‐year‐old nulliparous woman diagnosed with FIGO stage IB1 poorly differentiated (Grade 3) HPV‐associated mucinous cervical adenocarcinoma with signet‐ring cell features. Immunohistochemical evaluation demonstrated diffuse p16 overexpression, consistent with HPV‐associated disease (Figure 1). In addition, HPV genotyping was performed and confirmed the presence of high‐risk HPV genotype 16, further supporting the HPV‐related etiology of the tumor. These findings excluded gastric‐type adenocarcinoma, an HPV‐independent subtype known to exhibit a more aggressive clinical course and poorer prognosis.

The diagnosis was established following cervical conization, which revealed a 5.5‐mm invasive lesion with LVSI and clear margins. Pelvic magnetic resonance imaging (MRI) demonstrated no evidence of parametrial invasion or lymph node involvement.

Given the unusual histologic features, a comprehensive evaluation was performed to exclude the possibility of metastatic disease from another primary site, including gastroscopy, colonoscopy, and breast imaging, all of which were negative.

After extensive multidisciplinary counseling, including detailed discussion of oncologic risks and fertility considerations, the patient elected to pursue fertility‐sparing management. The patient provided written informed consent for conservative treatment, acknowledging the potential oncologic risks and reproductive implications. In addition, institutional ethical approval and written informed consent for publication of this case report were obtained.

She subsequently underwent radical vaginal trachelectomy with bilateral laparoscopic SLN mapping using ICG(Supporting Information: Video S1). Sentinel lymph nodes were successfully identified bilaterally and submitted for intraoperative frozen section analysis, which demonstrated no evidence of metastasis. In accordance with emerging evidence supporting the safety of SLN mapping in early cervical cancer (particularly findings from the SENTICOL and SENTIX studies [10–12]) and given the bilateral detection of negative sentinel nodes, complete pelvic lymphadenectomy was not performed.

During surgery, intraoperative frozen sections of the proximal cervical margin were obtained, confirming that the endocervical margin was free of malignant cells, allowing safe preservation of the uterine body.

Following completion of the trachelectomy, an intra‐abdominal cervical cerclage was placed using a cardiac band, positioned at the level of the uterine isthmus to provide structural support for future pregnancy.

Final histopathologic examination confirmed the diagnosis of HPV‐associated mucinous cervical adenocarcinoma with signet‐ring cell features, with negative surgical margins and negative SLNs on ultrastaging.

The postoperative course was uneventful.

The patient has been maintained under strict oncologic surveillance, including regular pelvic examinations, cervical cytology, and imaging. At 24 months of follow‐up, she remains clinically and radiologically disease‐free. Although she initially postponed pregnancy for personal reasons, she has received counseling regarding assisted reproductive technologies, considering the anatomical modifications resulting from radical trachelectomy.

While the oncologic outcome thus far is favorable, longer follow‐up remains necessary, particularly given the rare histologic subtype and the limited long‐term data regarding fertility‐sparing treatment in this context.

## 3. Discussion

Radical trachelectomy is an established fertility‐sparing surgical option for carefully selected patients with early‐stage cervical cancer, particularly those with FIGO stage IA1–IB1 disease and tumors ≤2 cm, in the absence of nodal involvement and other high‐risk features. Current international guidelines support fertility‐preserving surgery in selected patients who desire future childbearing, provided that oncologic safety is not compromised [1, 4].

Historically, radical hysterectomy with pelvic lymphadenectomy represented the standard treatment for early cervical cancer. However, increasing evidence has supported treatment de‐escalation in carefully selected patients. The SHAPE trial demonstrated that simple hysterectomy provides oncologic outcomes comparable to radical hysterectomy in low‐risk tumors ≤2 cm, highlighting the evolving paradigm toward less radical surgery in early‐stage cervical cancer [6]. In patients seeking fertility preservation, radical trachelectomy combined with lymph node assessment remains an accepted approach [3, 7, 8].

Accurate lymph node staging remains a critical component of surgical management. Over the past decade, SLN mapping has emerged as a reliable strategy to reduce morbidity associated with systematic lymphadenectomy while maintaining high diagnostic accuracy. The SENTICOL‐1 study demonstrated that bilateral negative sentinel nodes accurately predict the absence of nodal metastasis in early cervical cancer [10]. Subsequently, the SENTICOL‐2 randomized trial confirmed that SLN biopsy significantly reduces surgical morbidity without compromising oncologic safety when compared with full pelvic lymphadenectomy [11]. More recently, the SENTIX international prospective study further supported the safety of SLN mapping alone in patients with negative sentinel nodes, reporting favorable oncologic outcomes and reinforcing its role as a staging technique in early cervical cancer [12]. In the present case, bilateral SLN detection and negative intraoperative assessment supported the decision to avoid completion lymphadenectomy.

Fertility‐sparing surgery in cervical cancer has demonstrated favorable oncologic outcomes. Reported recurrence rates following radical trachelectomy range from 2.4% to 5.2%, with no significant differences in overall survival compared with radical hysterectomy in appropriately selected patients [7, 8]. Furthermore, evidence suggests that minimally invasive and open approaches yield comparable oncologic outcomes in early‐stage disease [5].

Reproductive outcomes following radical trachelectomy are encouraging. Clinical pregnancy rates range from 25.7% to 73%, with live birth rates reported between 63% and 68% among women attempting conception [9, 13, 15]. Vaginal radical trachelectomy is generally associated with higher pregnancy rates (reported up to 67.5%), while abdominal approaches tend to demonstrate lower fertility outcomes [7, 16]. Approximately 20%–21% of patients require assisted reproductive technologies, often due to complications such as cervical stenosis or altered cervical anatomy [13].

Despite these encouraging results, pregnancy after radical trachelectomy remains high‐risk. The most common obstetric complications include cervical insufficiency, second‐trimester miscarriage, preterm premature rupture of membranes, and preterm birth, with reported preterm delivery rates ranging from 37% to 44% [13–15]. Prophylactic cervical cerclage at the time of trachelectomy is therefore widely recommended, and close surveillance during pregnancy is essential. Cesarean delivery is generally advised due to altered cervical anatomy and potential risks of uterine rupture or infection [14].

An important aspect of this case is the rare histologic subtype. Cervical adenocarcinomas with signet‐ring cell features are extremely uncommon, and most reported cases represent metastases from gastrointestinal or breast primaries. For this reason, extensive investigation was performed in our patient to exclude other primary tumors before proceeding with fertility‐sparing treatment. Furthermore, HPV genotyping confirmed the presence of HPV‐16, supporting the diagnosis of HPV‐associated cervical adenocarcinoma and excluding gastric‐type adenocarcinoma, an HPV‐independent entity associated with a poorer prognosis and typically not considered suitable for conservative management.

Finally, although the patient remains disease‐free at 24 months, longer follow‐up is required. As highlighted in recent systematic reviews, the majority of recurrences after fertility‐sparing surgery occur within the first 3 years, emphasizing the importance of continued surveillance in these patients [8].

## 4. Conclusion

This case demonstrates the feasibility of fertility‐preserving surgery in a young woman with early‐stage HPV‐associated cervical adenocarcinoma exhibiting signet‐ring cell features, a rare histologic presentation.

The combination of radical vaginal trachelectomy and SLN mapping using ICG enabled uterine preservation while maintaining adequate oncologic staging and clear surgical margins.

As treatment strategies continue to evolve toward less radical surgical approaches in carefully selected patients, individualized decision‐making, comprehensive counseling, and multidisciplinary management remain essential to optimize both oncologic safety and reproductive outcomes.

## Funding

No funding was received for this manuscript.

## Disclosure

This case report was first presented, with great honor, at the 26th ESGO Congress in Rome, 2025, as an E‐poster and published in the official proceedings of the congress. It addresses a highly relevant and current topic: fertility preservation in a patient with cervical cancer. The abstract can be accessed via the following link: https://www.international-journal-of-gynecological-cancer.com/article/S1048-891X(24)21383-9/abstract.

## Conflicts of Interest

The authors declare no conflicts of interest.

## Supporting Information

Additional supporting information can be found online in the Supporting Information section.

## Supporting information


**Supporting Information** Video 1. Case report early cervical cancer. This video demonstrates a conservative surgical strategy in a patient with HPV‐associated mucinous adenocarcinoma of the cervix, FIGO stage IB1. Preserving reproductive potential without compromising oncologic safety.

## Data Availability

Data sharing is not applicable to this article, as no datasets were generated or analyzed during the current study.
